# Fasting Glycemia, Glycosylated Hemoglobin and Malnutrition Inflammation Are Associated With Sarcopenia in Older People With Chronic Kidney Disease Undergoing Hemodialysis Treatment: A Cross-Sectional Study

**DOI:** 10.7759/cureus.74432

**Published:** 2024-11-25

**Authors:** Mauricio Álvarez, Rosario Negrón, Carolina Neira-Maldonado, Felipe Ponce-Fuentes, Iván Cuyul-Vásquez

**Affiliations:** 1 Nephrology, Unidad de Diálisis, Hospital Dr Mauricio Heyermann Torres, Angol, CHL; 2 Physical Therapy, Universidad Mayor, School of Kinesiology, Faculty of Medicine and Health Sciences, Temuco, CHL; 3 Department of Therapeutic Processes, Faculty of Health Sciences, Universidad Católica de Temuco, Temuco, CHL

**Keywords:** elderly, end-stage kidney disease, hemodialysis, physical function, sarcopenia

## Abstract

Background:Contradictory data are available on the possible association between sarcopenia and other clinical disorders in patients with chronic kidney disease (CKD) undergoing hemodialysis.

Objective:To determine the association between sarcopenia and markers associated with systemic inflammation, fasting glycemia, and quality of life in older people with CKD undergoing hemodialysis.

Methods: This was an analytical cross-sectional study. People over 60 years with stage 5 CKD undergoing hemodialysis were invited to participate. Sarcopenia was assessed using the criteria of the European Working Group on Sarcopenia in Older People. Clinical variables such as body mass index, comorbidities, malnutrition inflammation, quality of life, fasting glycemia, glycosylated hemoglobin, hematocrit, uremia, creatinine, sodium, calcium, potassium, albumin, ferritin, C reactive protein were measured.

Results: Twenty-three patients with CKD were included. The prevalence of sarcopenia was 56.5%. Sarcopenic participants showed higher fasting glycemia, glycosylated hemoglobin, and malnutrition inflammation, as well as expected lower physical performance compared to nonsarcopenic participants. Correlations that ranged from moderate to strong were observed between sarcopenia and clinical characteristics such as fasting glycemia (r=0.48; P<0.05), glycosylated hemoglobin (r=0.44; P<0.05), malnutrition inflammation (r=0.46; P<0.05), skeletal muscle mass (r=-0.70; *P*<0.01), and balance capacity (r=-0.66; *P*<0.01). Regression analyses showed that fasting glycemia increased the risk of sarcopenia by 1.82 times (OR=1.82; *P*=0.04).

Conclusions: In a sample of older people with CKD who underwent hemodialysis, sarcopenia was correlated with fasting glycemia, glycosylated hemoglobin, and malnutrition inflammation. However, only fasting glycemia was found to be a significant predictor of sarcopenia. More studies are needed to determine the influence of glycemic control on sarcopenia in older people with CKD.

## Introduction

By 2040, chronic kidney disease (CKD) is predicted to become the fifth leading cause of death worldwide [1]. CKD patients who undergo hemodialysis are at increased risk of developing several complications, including infection, metabolic changes, and cardiovascular and bone disease [2]. High morbidity and mortality have been reported in patients with CKD, especially those with diabetes and hypertension [3]. The prevalence of CKD is increasing exponentially worldwide, with a prevalence of 14.2% in the United States (2007), and older people are a risk group [4]. In Chile, a CKD prevalence of 12% has been reported, with higher rates among women and older adults [5]. In older people, multiple co-occurring diseases may interact with kidney dysfunction to vary the prognosis [6]. Current evidence suggests that some interactions between CKD, sarcopenia, physical disability, and systemic inflammation may affect outcomes [7].

CKD can cause a significant loss of muscle mass and strength due to multiple interrelated mechanisms. Chronic inflammation, common in CKD, disrupts muscle protein balance, promoting muscle breakdown, and reducing synthesis of new proteins [8]. Furthermore, imbalances in electrolytes and hormonal levels, such as insulin resistance and vitamin D deficiency, can contribute to further muscle dysfunction [9]. The accumulation of uremic toxins can also negatively affect muscle function, reducing the ability of muscles to regenerate and maintain themselves [10]. These combined factors result in a progressive loss of muscle mass and strength, affecting the quality of life and functional capacity of patients with CKD [8].

A factor that promotes the occurrence and development of CKD is sarcopenia [11]. Sarcopenia is defined by the European Working Group on Sarcopenia in Older People as the presence of low skeletal muscle mass and low muscle function (strength or performance) [12]. The Third National Health and Nutrition Examination Survey (NHANES III, 2007) conducted in the United States (2007) included 13,770 people and found that the prevalence of sarcopenia among hemodialysis patients is around 50%, muscle wasting progresses as kidney function declines [4]. In this sense, abnormal age-related changes lead to muscle wasting, determining hormonal, myocellular, and immunologic modifications that are expressed as nutritional deficiency, metabolic acidosis, vitamin D deficiency, mineral bone disorders, insulin resistance, proteinuria, and chronic inflammation [13]. Therefore, sarcopenia results from complex mechanisms including protein imbalance, oxidative stress, inflammation, and uremic toxins [14]. Meta-analyses reveal that low muscle strength, mass, and physical performance are linked to higher mortality risk in CKD patients, with diagnosed sarcopenia also increasing mortality risk in dialysis patients [15]. In this sense, sarcopenia may lead to physical disability, low quality of life, and psychological distress in CKD patients [16]. However, evidence for associations with hospitalization and end-stage kidney disease progression is currently limited. Despite active research, measures for treatment and prevention remain challenging due to comorbidities and age-related factors [17]. Early recognition and effective interventions are crucial to mitigate complications associated with muscle wasting in CKD patients. It is essential to identify the biological, functional, and psychological markers that may be associated with sarcopenia in older people with CKD.

Although sarcopenia is a well-recognized disease in the older population, CKD per se is considered a risk factor for musculoskeletal disorders, mainly due to its associated metabolic disturbances and systemic inflammation [18]. However, contradictory data are available on the possible association between sarcopenia and other clinical disorders (e.g., systemic inflammation, diabetes mellitus) on CKD hemodialysis [11,19]. Therefore, the objective of this study was to determine the prevalence of sarcopenia and the differences between sarcopenic and non-sarcopenic patients, and explore the association between sarcopenia and a variety of markers associated with systemic inflammation, fasting glycemia, and quality of life in older people with CKD who undergo hemodialysis treatment. In the present study, we evaluated skeletal muscle mass and strength in older people with CKD who underwent hemodialysis to determine the prevalence of sarcopenia in a captive population. We expected that sarcopenia was common in older people with CKD in hemodialysis treatment, and we hypothesized that inflammation and glycemic markers may be associated with sarcopenia in this population.

## Materials and methods

Study design

An analytical cross-sectional study was designed for older people with stage 5 CKD in the hemodialysis unit of a single public hospital in Chile. The hemodialysis unit comprised 18 stations, each equipped with a single armchair and a dialysis machine (4008 S; Fresenius Medical Care, Rio de Janeiro, Brazil) located within a spacious area of 200 m². The hemodialysis unit operated in two shifts, with each shift lasting four hours (7:30 am - 12:30 pm and 12:30 pm - 4:30 pm). Each patient was scheduled for three sessions per week, each lasting four hours. The healthcare professionals assigned to each shift included two physicians, three nurses, three nurse assistants, one physical therapist, and one nutritionist.

Participants

A total of 40 patients with stage 5 CKD over 60 years old and dependent on a hemodialysis unit at a single hospital were eligible and invited to participate in the study. Only patients with stage 5 CKD were included in this study because the dialysis unit of this hospital performs kidney replacement therapy by hemodialysis following the recommendations of the Kidney Disease Improving Global Outcomes (KDIGO) 2024 Clinical Practice Guideline [20]. Exclusion criteria were as follows: patient with a metal prothesis or osteosynthesis, pacemaker, or other electronic device implanted in the body. Furthermore, older people with noncontrolled cardiovascular disease (e.g., unstable angina, cardiac arrhythmia, hypotension/hypertension, and diabetes mellitus), severe aortic stenosis, recent fracture/sprain/muscle strain, or visual or hearing or cognitive impairment that affects their ability to complete questionnaires or perform performance tests were excluded from participation. A convenience sampling was carried out through recruitment conducted in July 2023 by a physician not involved in the study. The sample size was composed of older people with CKD in kidney replacement therapy by hemodialysis who consented to participate voluntarily in the study. 

Measures

Participants were asked to provide a range of data, including demographic data, plasma samples, physical performance tests, and self-reported measures. In the first week of July 2023, the authors constructed a demographic survey and, with questionnaires, administered it to the participants by the team of healthcare professionals. The variables included age (years), sex (male or female), time on dialysis (in months), presence of comorbidities (no or yes), malnutrition inflammation status (MIS score, in points) and quality of life (Edmonton Symptom Assessment System - ESAS, in points). In the second week of July 2023, laboratory testing was conducted to obtain plasmatic levels. The blood test was fasting in the morning and the variables extracted from the participant's laboratory medical registry were fasting glycemia (mg/dL), glycosylated hemoglobin (%), glucose >200 (no or yes), glycosylated hemoglobin >7.5 (no or yes), hematocrit (%), uremia (mg/dL), creatinine (mg/dL), sodium (mg/dL), calcium (mg/dL), potassium (mg/dL), albumin (mg/dL) ferritin (mg/dL), and protein C-reactive protein (CRP). On a morning in the third week of July 2023, the physical performance was evaluated by the team of healthcare professionals through physical performance (Short Physical Performance Battery, SPPB), nutritional status (body mass index, BMI), and muscle mass (Lean Tissue Index, LTI) through anthropometry and bioimpedance.

Dependent variable: prevalence of sarcopenia

Sarcopenia was defined using the updated criteria of the European Working Group on Sarcopenia in Older People (EWGSOP2) [12]. To comply with the operational definition of sarcopenia according to EWGSOP2, it is necessary to demonstrate the presence of low skeletal muscle mass for age, measured by LTI, in conjunction with low muscle strength. Skeletal muscle mass was measured by LTI% and recorded by bioimpedance analysis (Body Composition Monitor, Fresenius Medical Care). Bioimpedance analysis is a non-invasive method for assessing body composition and clinical status [21]. The three-compartment bioimpedance analysis model expresses body composition as LTI, fat tissue index (FTI), and fluid overload [22]. LTI, calculated as lean tissue mass divided by height squared, is a consistent predictor of outcomes in patients with advanced kidney disease [22]. The procedure for bioimpedance analysis is carried out in upright position, typically involves connecting the subject to an impedance analyzer using a gel electrode on arms and legs, requiring technician placement and recumbent measurements [23]. Muscle strength was measured using the five-repetitions chair stand test, which is a reliable and valid measure of lower body strength in older adults [24]. It involves performing five sit-to-stand movements as quickly as possible, typically with arms folded across the chest [24]. The test has shown excellent intrarater, interrater, and test-retest reliability in various populations, and correlates with knee flexor muscle strength [25]. Physical performance was evaluated with the SPPB test. The SPPB is a well-established instrument for assessing physical performance in older adults [26]. It consists of three components: a timed 4-meter walk, repeated chair sit-to-stand test, and balance tests [26]. The SPPB uses a 12-point scoring system, with higher scores indicating better physical function [27]. Lower score in SPPB are associated with increased risk of disability and mortality, with scores below 10 indicating higher risk [27,28]. The SPPB has been shown to be a strong predictor of long-term disability, institutionalization, and all-cause mortality in community-dwelling older adults [27,28]. Participants were classified as sarcopenic, that is, lower skeletal muscle mass (low LTI) and lower muscle strength (high time in the chair test). Regarding the chair test, the values published by Bohannon in the elderly population were taken as reference values [29]. Those patients who also had a physical performance below 8 points in the SPPB were classified as 'severely sarcopenic' according to the definition stated above.

Independent variables

Independent variables were demographic data (age, sex, history of falls and time on hemodialysis) and clinical measures (BMI, comorbidities, MIS, ESAS-r, fasting glycemia, glycosylated hemoglobin, glucose, hematocrit, uremia, creatinine, sodium, calcium, potassium, albumin, ferritin, CRP, skeletal muscle mass and physical performance).

Data collection

In July 2023, assessors from the healthcare professionals’ team, who had undergone rigorous training, obtained demographic data, plasma samples, and physical performance data from all participants through face-to-face assessments. To promote reliability in the assessments and adherence to evaluation protocols, before undertaking the data recollection, all assessors underwent an eight-hour training period. Initially, the contents related to the outcome variables, constructs, instruments, and psychometric properties of each of the measurements were addressed theoretically (three teaching hours). Then, the practical component was carried out in order to develop skills in the application of the evaluation instruments and interpretation of the measurements obtained (three teaching hours). Finally, to certify the evaluators' skills, a practical test was carried out by measuring the primary outcomes (two teaching hours).

Data analysis

Continuous variables are presented as mean and standard deviation (x̄ ± SD), while categorical variables are presented as frequency (%). The prevalence of sarcopenia was calculated by dividing the total number of patients with sarcopenia by the total number of patients in the hemodialysis unit, multiplied by 100. The prevalence of sarcopenia is expressed as a percentage. The normality of the data was verified by the Shapiro-Wilk test. The comparison between sarcopenic and non-sarcopenic groups was made by Wilcoxon Mann Whitney test for non-parametric variables, and Student's T test for those with normal distribution. To assess the correlation and contribution of demographic, plasmatic and physical characteristics (independent variables) to the prevalence of sarcopenia in participants (dependent variable), a binary logistic regression was used. To define the variables to be included in the regression model, a Spearman correlation coefficient (r) was used to quantify the relationship between the prevalence of sarcopenia and the demographic of the participants (age, nutritional state, time on dialysis and malnutrition inflammation), plasmatic levels (fasting glycemia, glycosylated hemoglobin, hematocrit, uremia, creatinine, sodium, calcium, potassium, albumin, ferritin, and CRP), and physical variables (muscle mass, sitting to stand time and SPPB). The rank-biserial correlation was used to assess the association between the prevalence of sarcopenia and dichotomous categorical variables (i.e. sex, comorbidities presence, glucose >200 mg/dL, and glycosylated hemoglobin >7.5%). Independent variables were included in the models if the correlation with sarcopenia prevalence was significant (p<0.05). The strength of the association was classified as very strong correlation (r > 0.90), strong correlation (0.89 to 0.70), moderate correlation (0.69 to 0.50), weak correlation (0.49 to 0.30) and negligible correlation (r < 0.30) [30]. For all analyses, the significance level was set at 5%. Analyses were performed using IBM SPSS Statistics software version 25.0 (IBM Corp., Armonk, NY, USA).

Ethics approval

The present study protocol was reviewed and approved by the Scientific Ethics Committee of the Araucanía Sur Health Service, duly accredited by the Chilean Ministry of Health (registry number 145). All participants will give their informed consent. Deidentified data will be stored in a secure database with daily backup and hard copies of the data will be kept in an approved, secure storage facility.

## Results

Demographic and clinical characteristics

Of a total of 40 older people with CKD on hemodialysis invited to participate of the study, 17 did not meet eligibility criteria and 23 accepted and were included in the study. The mean age was 69.1 ± 5.8 years and 52.1% were women. Furthermore, the mean time on hemodialysis of the participants was 20.9 ± 28.2 months. Regarding clinical characteristics, 43.5% of the participants had a normal BMI. All participants had comorbidities. Regarding this, 100.0%, 91.3%, 73.9 and 56.5% of the participants had arterial hypertension, diabetes mellitus, dyslipidemia and depression, respectively. Furthermore, the values of fasting glycemia (229.4 ± 170.3 mg/dL), glycosylated hemoglobin (7.1 ± 1.8 %) and uremia (116.8 ± 30.0 mg/dL) values were high. Table 1 includes a summary of the demographic and clinical characteristics of the participants.

**Table 1:** Comparison of demographic and clinical characteristics between participants of sarcopenic and non-sarcopenic older people MIS, malnutrition inflammation; ESAS, Edmonton Symptom Assessment System; BMI, body mass index; CRP, C-reactive protein; a balance SPPB domain; b gait velocity SPPB domain; c chair stand capacity SPPB domain; d total SPPB score; P sarcopenic versus non-sarcopenic difference; *P < 0.05, **P < 0.01. Source: Prepared by the authors based on the results of the study.

Variable	Total (n = 23)	Sarcopenic (n = 13)	Non-sarcopenic (n = 10)	P
Demographic				
Age, years (mean ± SD)	69.1 ± 5.8	68.6 ± 5.2	69.7 ± 6.7	0.660
Sex, M/F	11/12	5/8	6/4	0.310
High BMI, n (%)	12 (43.5)	6/13 (46.2)	4/10 (40.0)	0.180
Previous fall presence (< 6 months), n (%)	2 (8.7)	2 (15.4)	0 (0.0)	0.190
Dialysis time, months (mean ± SD)	20.8 ± 28.1	27.9 ± 35.7	11.8 ± 8.8	0.800
Current Comorbidities				
Comorbidities number (mean ± SD)	4.1 ± 1.1	3.8 ± 1.3	4.2 ± 1.0	0.384
Arterial hypertension, n (%)	23 (100.0)	13 (100.0)	10 (100.0)	1.000
Diabetes mellitus, n (%)	21 (91.3)	12 (92.3)	9 (90.0)	0.850
Dyslipidemia, n (%)	17 (73.9)	10 (76.9)	7 (70.0)	0.710
Chronic heart failure, n (%)	5 (21.7)	3 (23.1)	2 (20.0)	0.860
Depression, n (%)	13 (56.5)	9 (69.2)	4 (40.0)	0.160
MIS, points (mean ± SD)	4.4 (2.7)	5.3 (3.1)	3.1 (1.4)	0.033^*^
ESAS, points (mean ± SD)	24.2 ± 18.4	29.1 ± 17.8	17.1 ± 17.6	0.110
Plasmatic Levels				
Fasting glycemia, mg/dL (mean ± SD)	229.4 ± 170.3	284.1 ± 208.2	158.2 ± 55.8	0.030^*^
Glycosylated hemoglobin, % (mean ± SD)	7.1 ± 1.8	7.7 ± 1.8	6.2 ± 1.4	
Glucose >200 mg/dL, n (%)	11 (47.8)	9/13 (69.2)	2/10 (20.0)	0.020^*^
Glycosylated hemoglobin >7.5%, n (%)	10 (43.5)	8/13 (61.5)	2/10 (20.0)	0.046^*^
Hematocrit, % (mean ± SD)	32.2 ± 5.3	31.8 ± 5.6	32.8 ± 5.0	0.760
Uremia, mg/dl (mean ± SD)	116.8 ± 30.0	108.4 ± 24.7	127.8 ± 33.9	0.150
Creatinine, mg/dl (mean ± SD)	6.0 ± 2.3	5.4 ± 1.9	6.9 ± 2.5	0.130
Sodium (mEq/L)	135.7 ± 3.5	135.8 ± 4.4	135.6 ± 2.2	0.910
Calcium (mg/dL)	9.7 ± 0.8	9.5 ± 0.7	9.9 ± 1.0	0.360
Potassium (mEq/L)	4.5 ± 0.6	4.5 ± 0.5	4.5 ± 0.8	0.810
Albumin (g/dL)	3.9 ± 0.4	3.9 ±0.5	3.9 ± 0.5	0.750
Ferritin (ng/ml)	380.0 ± 268.2	428.3 ± 328.4	317.1 ± 156.0	0.660
CRP (mg/dL)	11.1 ± 17.8	11.1 ± 20.8	11.0 ± 14.1	0.990
Physical status				
Muscle mass (kg)	10.3 ± 2.0	9.2 ± 1.5	11.8 ± 1.6	0.001^**^
Balance ^a^, (points)	2.5 ± 1.4	1.8 ± 1.3	3.5 ± 0.7	0.001^**^
Gait velocity ^b^, sec (mean ± SD)	4.4 ± 3.2	4.9 ± 4.0	5.2 ± 2.3	0.862
5-rep chair stand test ^c^, sec (mean ± SD)	8.7 ± 6.4	11.4 ± 2.3	6.7 ± 7.7	0.080
Physical performance ^d^, (mean ± SD)	7.2 ± 4.1	4.7 ± 3.5	10.5 ± 1.8	0.000^**^

Sarcopenia prevalence and differences between sarcopenic and non-sarcopenic participants

The prevalence of sarcopenia in the total sample was 56.5%. No significant differences were found in demographics and comorbidities between sarcopenic and non-sarcopenic patients (P > 0.05). Fasting glycemia (284.1 ± 208.2 mg/dL; CI95% = -267.2 - 15.4) and glycosylated hemoglobin (7.7 ± 1.8%; CI95% = -3.0 - -0.1) in sarcopenic participants were significantly higher than those of non-sarcopenic participants (P < 0.05). In addition, the glucose >200mg/dL (9/13 = 69.2%) and glycosylated hemoglobin >7.5% (8/13 = 61.5%) were significantly more frequent in participants than in non-sarcopenic participants (P < 0.05). The differences in skeletal muscle mass (2.6 ± 0.6 kg; CI95% = 1.3 - 4.0), balance (1.7 ± 0.5; CI95% = 0.8 - 2.7) and physical performance (5.8 ± 1.2 points; CI95% = 3.3 - 8.3) were significantly lower in participants with sarcopenia than in non-sarcopenic participants (P < 0.01). Malnutrition inflammation (5.3 ± 3.1 points; CI95% = -4.2 - -0.2) was significantly higher in sarcopenic participants than in non-sarcopenic participants (P < 0.01). No differences were found in quality of life and other hematological and biochemical markers between sarcopenic and non-sarcopenic participants (P > 0.05). The clinical characteristics between sarcopenic and non-sarcopenic older people with CKD are shown in Table 1.

Sarcopenia-associated clinical characteristics

The correlation coefficients showed a significant moderate positive correlation between sarcopenia with fasting glycemia (r = 0.48; P < 0.05), glycosylated hemoglobin (r = 0.44; P < 0.05), glucose >200 (r = 0.49; P < 0.05), glycosylated hemoglobin >7.5 (r = 0.42; P < 0.05), malnutrition inflammation (r = 0.46; P < 0.05). Furthermore, a significant strong negative correlation was established between sarcopenia with muscle mass (r = -0.70; P < 0.01) and balance (r = -0.66; P < 0.01). Furthermore, the binary logistic regression model that examined the predictors of sarcopenia found that in participants in CKD with fasting hyperglycemia, the risk of sarcopenia increases almost two times (OR = 1.82; 95% CI= 1.00 - 1.03; P = .048). These results suggest that fasting glycemia is strongly associated with the prevalence of sarcopenia among older people with CKD in hemodialysis treatment. See Table 2 and Table 3 for details of the analysis.

**Table 2:** Correlation coefficients between sarcopenia and clinical characteristics MIS, Malnutrition-Inflammation Score; *P < 0.05, **P < 0.01. aSpearman’s correlation coefficient; bRank biserial correlation. Bold, 'strong correlation'. Source: Prepared by the authors based on the results of the study.

Independent Variables Dependent Variable	Fasting glycemia (mg/dL) ^a^	Glycosylated hemoglobin (mg/dL) ^a^	Glucose >200 ^b^	Glycosylated hemoglobin >7.5 ^b^	MIS total (points)^a^	Muscle mass (kg) ^a^	Balance (points) ^a^
Sarcopenia	0.48^*^	0.44^*^	0.49^*^	0.42^*^	0.46^*^	-0.70^**^	-0.66^**^

**Table 3:** Logistic regression analysis of clinical characteristics associated with sarcopenia B, Coefficient Estimation; SE, Standard Error; P, P-value; β, Beta Coefficient; CI, Confidence Interval; LB, Lower Border; UB, Upper Border. *P < 0.05. Source: Prepared by the authors based on the results of the study.

Variable	B	SE	P	β	LB CI_95%_ β	UB CI_95%_ β
Constant	-2.574	1.425	0.071	0.076	-	-
Age	0.016	0.108	0.881	0.984	0.797	1.215
Fasting glycemia	0.014	0.048	0.048^*^	1.815	1.000	1.029
Glucose >200 mg/dL	-1.014	1.785	0.570	0.363	0.011	11.992
Glycosylated hemoglobin	-0.221	0.634	0.727	0.802	0.231	2.779

## Discussion

The objectives of this study were to determine the prevalence of sarcopenia and the differences between sarcopenic and non-sarcopenic, and determine the association between sarcopenia and a variety of markers associated with systemic inflammation, fasting glycemia, and quality of life in older people with CKD undergoing hemodialysis treatment. In this context, the prevalence of sarcopenia was 56.5% in this sample. Furthermore, sarcopenic participants showed higher fasting glycemia, glycosylated hemoglobin, and malnutrition inflammation, as well as expected lower physical performance compared to non-sarcopenic participants. Correlations that ranged from weak to strong were observed between the presence of sarcopenia and clinical characteristics such as fasting glycemia, glycosylated hemoglobin, malnutrition inflammation, skeletal muscle mass and balance capacity. Finally, our regression analyses showed that fasting glycemia showed a strong association with sarcopenia; and increased the risk of sarcopenia two times.

Sarcopenia prevalence

The prevalence of 56.5% reported in our study is consistent with those of previous studies that examined the prevalence of sarcopenia in patients with CKD undergoing hemodialysis treatment [31-36]. Recently, the Duarte et al. (2024) global systematic review and meta‐analysis reported a global prevalence of sarcopenia of 24.5% in patients with CKD [35], and ranged from 6.7% [31] to 82.5% [36] in participants in hemodialysis treatment diagnosed according to the EWGSOP2 criteria [12]. Our study is comparable to other similar studies in Latin America. For example, Abdala et al. (2021) report a prevalence of sarcopenia at 16% in Argentinian patients with CKD [31], however, this sample was youngest (55.6 versus 69.1 years), and the methods used to measure muscle strength were different compared to our participants (handgrip versus chair stand test). It is possible that the older people in our study have more advanced ageing changes, and the difference in the muscle strength assessment method contributed to the high prevalence of sarcopenia in our CKD participants. Lamarca et al. (2014) reported that Brazilian patients with CKD on hemodialysis treatment, a prevalence of sarcopenia of 63.7% has been reported [33]. Despite the similar age of the participants, the higher prevalence in our study may be due to methodological differences in skeletal muscle mass assessments (dual-energy X-ray absorptiometry versus bioelectrical impedance analysis). Zhou et al. (2022) in 3,196 participants [32] and Zhou et al. (2023) in 2,743 participants [34], are the largest studies investigating the prevalence of sarcopenia in participants with CKD in hemodialysis treatment; they found a prevalence of 36.2% and 15.6%, respectively. These two large studies followed the Asian Work Group on Sarcopenia (AWGS) guideline for sarcopenia diagnosis, which implies measuring muscle strength with handgrip dynamometry. On the other hand, according to the recommendations of EWGSOP2, the muscle strength in our study was measured with the chair stand test, which implies a more vigorous physical effort compared to handgrip dynamometry. These significant differences may partially explain the higher prevalence of sarcopenia in our study compared to those two previous large studies in patients with CKD. 

Differences between sarcopenic and non-sarcopenic patients

Our study did not show differences in age, sex, time on hemodialysis, nutritional status, presence of comorbidities, hematocrit, creatinine, calcium, albumin, and CRP between CKD-sarcopenic and non-sarcopenic patients on hemodialysis treatment. This is in line with previous studies [11,19,31,37]. However, the differences between sarcopenic and non-sarcopenic participants with CKD on hemodialysis treatment are controversial with respect to glycemic plasmatic levels in previous studies [11,19]. In this sense, Formiga et al. (2022) did not observe significant differences in fasting plasma glucose and glycosylated hemoglobin between sarcopenic and non-sarcopenic patients with CKD on hemodialysis treatment [19]. Similarly, Yu et al. (2021) in non-dialytic and diabetes mellitus CKD adults did not observe significant differences in fasting plasma glucose between sarcopenic and non-sarcopenic patients [11]. Regarding this, we think that this difference may be determined by the high prevalence of diabetes mellitus in the population studied (91%). When reviewing the indicators, we can observe poor metabolic control of the disease, which is recognised to be associated with micro-inflammatory states that cause sarcopenia [38]. An additional explanation for these results may be the levels of physical activity, sedentary behaviour, diet, and educational level. However, these variables were not considered in our study.

Sarcopenia-associated clinical characteristics

In our study, we did not find a significant association between the prevalence of sarcopenia and the presence of current and demographic comorbidities, contrary to the findings of previous studies. Formiga et al. (2022) in their large cross‑sectional study in elderly patients with CKD showed that sarcopenia is associated with higher age, male sex, absence of hypertension as a comorbidity, poor cognitive performance, and lower BMI [19]. Variations in the prevalence of diabetes mellitus across studies may account for the conflicting results. In our study, 91.3% of older adults with CKD had a prior diagnosis of diabetes mellitus. In contrast, diabetes mellitus was reported in only 22.2% and 36.6% of participants in the studies by Yu et al. (2021) and Formiga et al. (2022), respectively. Despite the high prevalence of diabetes among our participants, we did not observe a significant association with sarcopenia. This finding contrasts with Lee et al. (2020), who identified a significant link between sarcopenia and diabetes in older adults with CKD undergoing hemodialysis therapy [39]. A potential explanation could be that both diabetes and CKD involve chronic inflammation, which may impair muscle metabolism over time [40].

Furthermore, in our study, fasting glycemia and glycosylated hemoglobin were significantly associated with the prevalence of sarcopenia. Poor glycemic control in patients with CKD treated with hemodialysis in our study could partially explain the lower muscle mass, balance capacity and physical performance in sarcopenic patients. In fact, hyperglycemia has been associated with less muscle quality [41]. Furthermore, insulin resistance increases with age and is the basis of several mechanisms in the induction of sarcopenia. In this sense, insulin is an anabolic hormone that stimulates protein synthesis, including those present in muscles. Thus, defects in insulin signaling can lead to reduced muscle synthesis [5].

The present study found a strong association between the presence of sarcopenia and physical status in older adult participants with CKD on hemodialysis therapy; this is in line with previous studies. Yuenyongchaiwat et al. (2021) reported a moderate negative correlation between the presence of sarcopenia with the mass of the skeletal muscle, the strength of the handgrip, and the speed of gait [42]. Sarcopenia, the loss of skeletal muscle mass and its functionality, is highly prevalent in patients with CKD and is strongly associated with higher morbidity and mortality [43]. Patients with sarcopenia gradually lose muscle mass and strength, while the degree of sarcopenia is associated with the stage of CKD, especially in men [44]. In older adults, where sarcopenia is even more frequent due to the impact of ageing, less physical activity, and the more prevalent end stage of CKD, sarcopenia is even more profound and is refractory most of the time. Future studies should explore the implementation of physical and nutritional interventions in the prevention and treatment of sarcopenia in older adult patients with CKD on hemodialysis.

Strength and limitations

Our study had several strengths. It was performed using comprehensive evaluations, including the availability of body composition, plasmatic and physical markers. In addition, our cohort included hemodialysis patients as a specific population, in which the characteristics associated with the sarcopenia presence have not yet been fully elucidated in CKD patients. Despite the strengths, the main limitations of our study were its cross-sectional design, use of a single hospital, and relatively small sample size. In addition, the levels of physical activity, sedentary behavior, diet and educational level were not considered in our study. In this sense, it might be difficult to establish a causal relationship of sarcopenia with CKD, and the prevalence of sarcopenia in hemodialysis patients in the study may have limited generalizability.

## Conclusions

In a sample of older people with CKD undergoing hemodialysis treatment, the presence of sarcopenia was correlated with fasting glycemia, glycosylated hemoglobin and malnutrition inflammation. However, only fasting glycemia proved to be a significant predictor of the presence of sarcopenia. More studies are necessary to determine the influence of glycemic control on sarcopenia in older people with CKD.
